# The Antiseptic “Flavine” (Acriflavine)

**Published:** 1918-01

**Authors:** 


### THE ANTISEPTIC "FLAVINE" (ACRIFLAVINE)

The following has been officially communicated to the press by the Medical Research Committee:

The incomplete statements which have appeared from time to time in the press with regard to the antiseptic "Flavine" have necessarily led to some misapprehension of the situation by the public. The impression has been created that flavine is a substance of magical potency which can cure infected wounds, and that there has been an unnecessary delay in making supplies of it available for general use. It should be realized that no antiseptic, even a theoretically ideal antiseptic when that is obtained, can ever be more than an adjunct to skilled and thorough surgery. Flavine must be thought of accordingly in relation to the other new antiseptics which have been brought into successful use during the course of the war.

These fall into two main classes. One depends upon the use of chlorine compounds, as in the well-known "Eusol" introduced by Edinburgh workers, the similar "Dakin" solution and the more recent chloramaine-T, introduced by Dr. Dakin from the Leeds laboratories. These have wide use in our armies, and are now known all over the world. The researches from which these arose, and which are still in progress, have all been supported from the beginning by government help through the Medical Research Committee. The second class, to which flavine belongs, includes the elaborate compounds of which most are brilliantly colored and are used as dyes. From the first month of war special searches have been made among those on behalf of the Research Committee for unrecognized antiseptics of high value. Work at Haslar and at the London Hospital for the committee showed very early the value of malachite green, and this is giving valuable routine results in naval hospitals and elsewhere. When it was desired to investigate Flavine, which was a little-known, but patented, German dye, none was available in this country, and its manufacture requires highly skilled and laborious work. The committee, however, caused a supply to be specially made in their biochemical department for investigation by Dr. Browning at the Bland-Sutton Institute. His remarks were first published in January, 1917. Three months previously, however, as a result of the first preliminary experiments, the committee arranged that preparation should be made for the difficult processes of commercial manufacture, and all the scientific information at their command was made freely available. As a result of this early action they were able to supplement their own supply in use for the first laboratory experiments and clincial trials by a large number of samples, which were sent in April to a selected group of consultant surgeons to the forces and other responsible surgeons for the purpose of securing official reports for the guidance of the Admiralty, the War Office and civilian institutions, whose action could not rest upon any single testimony or any small scale supply of the antiseptic. It is already clear that the special uses of Flavine for particular purposes have still to be carefully defined in relation to other antiseptics and to the operative methods of surgery, to which it can at best only be a valuable aid. While this concerted official study is in progress two or three firms are already preparing for commercial supply to the public. Under our patent law it has been necessary for them to procure license to manufacture from the Board of Trade. The hearing of applications for licenses has already taken place, and it is hoped that licenses to manufacture will very shortly be issued by the board. It is proposed that all the manufacturing firms shall use one and the same name for the substance, a condition which will restrain any firm from securing monopoly privileges by wide advertisement of a fancy name. The Medical Research Committee have proposed for important technical reasons that the substance shall be officially called "Acriflavine" in this country. This will avoid the German name "Trypaflavin," which is registered as a trade-mark, and prevent confusion with an existing vegetable dye already called "Flavine." No doubt the firms concerned will announce at the earliest moment their readiness to supply Acriflavine commercially.

During the progress of this work the committee have more recently put at Dr. Browning's disposal for study a compound closely allied to Acriflavine, which already appears to have identical or even superior properties, and if this be confirmed it will be easier to manufacture and cheaper for the public. This substance will be officially known as "Proflavine." An early scientific publication will be made on this subject.

The degree of government support which has been given from the beginning to the researches upon Acriflavine and other antiseptics has not always been made a matter of public knowledge. It is proper that financial and scientific help given officially should not diminish in any way the credit due to individual scientific workers or scientific institutions.—*Dental Record.*

